# Supplementary material for: Hot spot prediction in protein-protein interactions by an ensemble system
Source: BMC Syst Biol. 2018 Dec 31;12(Suppl 9):132. doi: 10.1186/s12918-018-0665-8 (PMC6311905; doi:10.1186/s12918-018-0665-8)
Supplement: Supplementary file 2 — All relevant MATLAB code. A simple Matlab implement of our predictor. (ZIP 19,742 kb) [file 12918_2018_665_MOESM2_ESM.zip › Additional file 2/Read ASA/ASA website/BID/1IHBB.html]

 NetSurfP - Protein Surface Accessibility and Secondary Structure Predictions

  

|  |  |  |
| --- | --- | --- |
|  |  | NetSurfP - Protein Surface Accessibility and Secondary Structure Predictions  Technical University of Denmark |

  


---

```
# For publication of results, please cite:
# A generic method for assignment of reliability scores applied to solvent accessibility predictions.
# Bent Petersen, Thomas Nordahl Petersen, Pernille Andersen, Morten Nielsen and Claus Lundegaard
# BMC Structural Biology 2009, 9:51 doi:10.1186/1472-6807-9-51
#
# Column 1: Class assignment - B for buried or E for Exposed - Threshold: 25% exposure, but not based on RSA
# Column 2: Amino acid
# Column 3: Sequence name
# Column 4: Amino acid number
# Column 5: Relative Surface Accessibility - RSA
# Column 6: Absolute Surface Accessibility
# Column 7: Z-fit score for RSA prediction
# Column 8: Probability for Alpha-Helix
# Column 9: Probability for Beta-strand
# Column 10: Probability for Coil
E M  1IHBB                  1    0.533 106.713  -1.307   0.003   0.003   0.994
E A  1IHBB                  2    0.261  28.707  -1.916   0.018   0.047   0.935
E E  1IHBB                  3    0.670 116.997  -1.068   0.058   0.017   0.925
E P  1IHBB                  4    0.574  81.436  -0.149   0.058   0.017   0.925
E W  1IHBB                  5    0.284  68.278  -1.001   0.109   0.005   0.886
E G  1IHBB                  6    0.264  20.808   0.808   0.181   0.016   0.803
B N  1IHBB                  7    0.061   8.901   0.334   0.278   0.093   0.628
B E  1IHBB                  8    0.027   4.804   0.910   0.701   0.107   0.192
B L  1IHBB                  9    0.022   4.047   1.049   0.911   0.033   0.057
B A  1IHBB                 10    0.086   9.433   0.638   0.938   0.007   0.055
B S  1IHBB                 11    0.092  10.818   1.198   0.975   0.003   0.022
B A  1IHBB                 12    0.019   2.094   0.906   0.938   0.007   0.055
B A  1IHBB                 13    0.029   3.240  -0.108   0.938   0.007   0.055
E A  1IHBB                 14    0.431  47.452   0.371   0.802   0.014   0.185
E R  1IHBB                 15    0.467 106.920   0.306   0.246   0.004   0.750
E G  1IHBB                 16    0.437  34.360  -0.807   0.016   0.005   0.979
B D  1IHBB                 17    0.142  20.419   0.313   0.053   0.005   0.942
E L  1IHBB                 18    0.279  51.048   1.021   0.879   0.010   0.111
E E  1IHBB                 19    0.322  56.201   0.813   0.911   0.033   0.057
B Q  1IHBB                 20    0.031   5.537   0.977   0.950   0.028   0.022
B L  1IHBB                 21    0.021   3.845   1.451   0.950   0.028   0.022
E T  1IHBB                 22    0.351  48.753   1.602   0.975   0.003   0.022
B S  1IHBB                 23    0.104  12.212   1.570   0.975   0.003   0.022
B L  1IHBB                 24    0.031   5.713   0.867   0.938   0.007   0.055
B L  1IHBB                 25    0.099  18.090   0.372   0.938   0.007   0.055
E Q  1IHBB                 26    0.447  79.763   0.387   0.802   0.014   0.185
E N  1IHBB                 27    0.235  34.389   0.843   0.406   0.004   0.590
E N  1IHBB                 28    0.342  50.040  -1.156   0.058   0.017   0.925
B V  1IHBB                 29    0.038   5.794   0.084   0.018   0.019   0.964
E N  1IHBB                 30    0.419  61.342   0.825   0.019   0.141   0.840
B V  1IHBB                 31    0.144  22.164   1.156   0.020   0.205   0.775
E N  1IHBB                 32    0.361  52.850   0.506   0.020   0.205   0.775
E A  1IHBB                 33    0.426  46.989   0.491   0.056   0.142   0.802
E Q  1IHBB                 34    0.553  98.712   0.446   0.052   0.084   0.864
E N  1IHBB                 35    0.341  49.966  -0.332   0.053   0.043   0.903
E G  1IHBB                 36    0.647  50.935  -1.216   0.058   0.017   0.925
E F  1IHBB                 37    0.535 107.435  -0.452   0.018   0.019   0.964
E G  1IHBB                 38    0.307  24.145  -0.474   0.016   0.005   0.979
B R  1IHBB                 39    0.219  50.243   0.884   0.181   0.016   0.803
B T  1IHBB                 40    0.059   8.169   0.519   0.386   0.097   0.517
B A  1IHBB                 41    0.022   2.457   1.261   0.751   0.050   0.199
B L  1IHBB                 42    0.023   4.193   0.903   0.911   0.033   0.057
B Q  1IHBB                 43    0.089  15.878   0.925   0.938   0.007   0.055
B V  1IHBB                 44    0.072  11.097   0.830   0.950   0.028   0.022
B M  1IHBB                 45    0.020   4.042   0.952   0.879   0.010   0.111
B K  1IHBB                 46    0.063  12.918  -1.062   0.802   0.014   0.185
E L  1IHBB                 47    0.402  73.625  -0.089   0.430   0.016   0.555
E G  1IHBB                 48    0.441  34.691  -0.636   0.053   0.005   0.942
B N  1IHBB                 49    0.115  16.777   0.293   0.053   0.005   0.942
E P  1IHBB                 50    0.293  41.633   1.160   0.879   0.010   0.111
E E  1IHBB                 51    0.294  51.292   0.918   0.911   0.033   0.057
B I  1IHBB                 52    0.038   7.104   0.936   0.911   0.033   0.057
B A  1IHBB                 53    0.025   2.733   1.304   0.950   0.028   0.022
E R  1IHBB                 54    0.335  76.623   1.584   0.950   0.028   0.022
B R  1IHBB                 55    0.111  25.373   1.646   0.950   0.028   0.022
B L  1IHBB                 56    0.035   6.409   0.901   0.938   0.007   0.055
B L  1IHBB                 57    0.111  20.287   0.465   0.879   0.010   0.111
E L  1IHBB                 58    0.363  66.429   0.088   0.802   0.014   0.185
E R  1IHBB                 59    0.270  61.738   1.220   0.339   0.016   0.645
E G  1IHBB                 60    0.336  26.412  -1.238   0.115   0.016   0.868
B A  1IHBB                 61    0.041   4.540  -0.195   0.053   0.043   0.903
E N  1IHBB                 62    0.397  58.106   0.598   0.052   0.084   0.864
B P  1IHBB                 63    0.207  29.444   1.034   0.056   0.142   0.802
E D  1IHBB                 64    0.382  55.104  -0.015   0.064   0.216   0.721
E L  1IHBB                 65    0.400  73.277   0.532   0.056   0.142   0.802
E K  1IHBB                 66    0.502 103.261   0.095   0.056   0.142   0.802
E D  1IHBB                 67    0.265  38.244  -0.484   0.053   0.043   0.903
E R  1IHBB                 68    0.640 146.560  -1.553   0.058   0.017   0.925
E T  1IHBB                 69    0.535  74.191  -0.304   0.018   0.019   0.964
E G  1IHBB                 70    0.270  21.288  -0.867   0.053   0.005   0.942
B F  1IHBB                 71    0.212  42.468   0.718   0.115   0.016   0.868
B A  1IHBB                 72    0.057   6.314   0.382   0.386   0.097   0.517
B V  1IHBB                 73    0.024   3.627   1.127   0.701   0.107   0.192
B I  1IHBB                 74    0.019   3.496   1.250   0.911   0.033   0.057
B H  1IHBB                 75    0.078  14.225   0.824   0.938   0.007   0.055
B D  1IHBB                 76    0.091  13.156   1.149   0.975   0.003   0.022
B A  1IHBB                 77    0.018   1.940   0.944   0.938   0.007   0.055
B A  1IHBB                 78    0.024   2.678   0.253   0.879   0.010   0.111
E R  1IHBB                 79    0.384  87.867   0.207   0.802   0.014   0.185
E A  1IHBB                 80    0.408  44.918   0.270   0.321   0.003   0.675
E G  1IHBB                 81    0.422  33.243  -0.852   0.016   0.005   0.979
B F  1IHBB                 82    0.136  27.335   0.385   0.109   0.005   0.886
E L  1IHBB                 83    0.283  51.744   0.961   0.879   0.010   0.111
E D  1IHBB                 84    0.296  42.668   0.713   0.911   0.033   0.057
B T  1IHBB                 85    0.028   3.925   1.124   0.911   0.033   0.057
B L  1IHBB                 86    0.025   4.596   1.281   0.950   0.028   0.022
E Q  1IHBB                 87    0.339  60.545   1.674   0.950   0.028   0.022
B T  1IHBB                 88    0.107  14.869   1.576   0.950   0.028   0.022
B L  1IHBB                 89    0.036   6.610   1.016   0.911   0.033   0.057
B L  1IHBB                 90    0.103  18.804   0.149   0.911   0.033   0.057
E E  1IHBB                 91    0.353  61.739  -0.027   0.717   0.014   0.269
E F  1IHBB                 92    0.261  52.302   0.976   0.339   0.016   0.645
E Q  1IHBB                 93    0.338  60.438  -1.144   0.115   0.016   0.868
B A  1IHBB                 94    0.049   5.444   0.124   0.053   0.043   0.903
E D  1IHBB                 95    0.429  61.776   0.465   0.056   0.142   0.802
B V  1IHBB                 96    0.181  27.743   1.111   0.064   0.216   0.721
E N  1IHBB                 97    0.350  51.284   0.279   0.125   0.227   0.648
E I  1IHBB                 98    0.459  84.915   0.741   0.118   0.150   0.732
E E  1IHBB                 99    0.539  94.216   0.568   0.113   0.087   0.800
E D  1IHBB                100    0.355  51.213  -0.175   0.113   0.043   0.844
E N  1IHBB                101    0.631  92.349  -1.216   0.115   0.016   0.868
E E  1IHBB                102    0.508  88.713  -0.070   0.058   0.017   0.925
E G  1IHBB                103    0.313  24.633  -0.650   0.018   0.019   0.964
B N  1IHBB                104    0.228  33.379   0.857   0.115   0.016   0.868
B L  1IHBB                105    0.061  11.224   0.421   0.278   0.093   0.628
B P  1IHBB                106    0.025   3.505   1.042   0.831   0.044   0.125
B L  1IHBB                107    0.022   4.047   0.962   0.879   0.010   0.111
B H  1IHBB                108    0.081  14.698   0.589   0.938   0.007   0.055
B L  1IHBB                109    0.087  15.893   0.978   0.975   0.003   0.022
B A  1IHBB                110    0.019   2.061   0.844   0.938   0.007   0.055
B A  1IHBB                111    0.025   2.755   0.110   0.938   0.007   0.055
E K  1IHBB                112    0.386  79.400   0.186   0.802   0.014   0.185
E E  1IHBB                113    0.434  75.767   0.199   0.321   0.003   0.675
E G  1IHBB                114    0.420  33.023  -0.932   0.016   0.005   0.979
B H  1IHBB                115    0.149  27.049   0.056   0.053   0.005   0.942
E L  1IHBB                116    0.287  52.586   0.743   0.802   0.014   0.185
E R  1IHBB                117    0.287  65.608   0.655   0.911   0.033   0.057
B V  1IHBB                118    0.032   4.965   0.863   0.911   0.033   0.057
B V  1IHBB                119    0.023   3.474   1.327   0.911   0.033   0.057
E E  1IHBB                120    0.332  57.948   1.563   0.950   0.028   0.022
B F  1IHBB                121    0.103  20.592   1.204   0.950   0.028   0.022
B L  1IHBB                122    0.029   5.365   1.038   0.950   0.028   0.022
B V  1IHBB                123    0.088  13.602   0.506   0.879   0.010   0.111
E K  1IHBB                124    0.421  86.559   0.702   0.717   0.014   0.269
E H  1IHBB                125    0.239  43.401   1.456   0.257   0.016   0.727
E T  1IHBB                126    0.348  48.240  -1.138   0.058   0.017   0.925
B A  1IHBB                127    0.078   8.629  -0.456   0.053   0.043   0.903
E S  1IHBB                128    0.500  58.612   0.082   0.056   0.142   0.802
E N  1IHBB                129    0.313  45.896   0.275   0.056   0.142   0.802
B V  1IHBB                130    0.155  23.900   0.938   0.113   0.087   0.800
E G  1IHBB                131    0.436  34.305   0.202   0.184   0.043   0.773
E H  1IHBB                132    0.479  87.166   0.763   0.184   0.043   0.773
E R  1IHBB                133    0.499 114.179   0.233   0.184   0.043   0.773
B N  1IHBB                134    0.259  37.947  -0.361   0.115   0.016   0.868
E H  1IHBB                135    0.616 112.123  -1.277   0.058   0.017   0.925
E K  1IHBB                136    0.489 100.587  -0.124   0.018   0.019   0.964
E G  1IHBB                137    0.211  16.606  -0.743   0.053   0.005   0.942
B D  1IHBB                138    0.220  31.673   0.957   0.181   0.016   0.803
B T  1IHBB                139    0.057   7.920   0.533   0.455   0.046   0.498
B A  1IHBB                140    0.024   2.601   1.088   0.879   0.010   0.111
B C  1IHBB                141    0.021   2.948   1.149   0.938   0.007   0.055
B D  1IHBB                142    0.112  16.211   0.825   0.975   0.003   0.022
B L  1IHBB                143    0.083  15.271   1.155   0.975   0.003   0.022
B A  1IHBB                144    0.017   1.851   0.912   0.938   0.007   0.055
B R  1IHBB                145    0.046  10.534   0.010   0.938   0.007   0.055
E L  1IHBB                146    0.440  80.582   0.145   0.802   0.014   0.185
E Y  1IHBB                147    0.490 104.798   0.279   0.246   0.004   0.750
E G  1IHBB                148    0.539  42.396  -1.258   0.016   0.005   0.979
B R  1IHBB                149    0.180  41.243   0.351   0.053   0.005   0.942
E N  1IHBB                150    0.388  56.862   1.205   0.802   0.014   0.185
E E  1IHBB                151    0.310  54.227   1.157   0.911   0.033   0.057
B V  1IHBB                152    0.027   4.135   1.133   0.911   0.033   0.057
B V  1IHBB                153    0.039   5.948   0.897   0.911   0.033   0.057
E S  1IHBB                154    0.421  49.306   1.767   0.950   0.028   0.022
B L  1IHBB                155    0.109  19.885   1.678   0.950   0.028   0.022
B M  1IHBB                156    0.049   9.865   1.152   0.950   0.028   0.022
B Q  1IHBB                157    0.187  33.452   0.556   0.879   0.010   0.111
E A  1IHBB                158    0.451  49.722   0.246   0.802   0.014   0.185
E N  1IHBB                159    0.304  44.491   1.260   0.430   0.016   0.555
E G  1IHBB                160    0.403  31.708  -1.838   0.115   0.016   0.868
B A  1IHBB                161    0.210  23.153  -1.352   0.018   0.019   0.964
E G  1IHBB                162    0.833  65.565  -0.876   0.003   0.003   0.994

Explain the output.  Go back.
```

---
